# Landscape context and the biophysical response of rivers to dam removal in the United States

**DOI:** 10.1371/journal.pone.0180107

**Published:** 2017-07-10

**Authors:** Melissa M. Foley, Francis J. Magilligan, Christian E. Torgersen, Jon J. Major, Chauncey W. Anderson, Patrick J. Connolly, Daniel Wieferich, Patrick B. Shafroth, James E. Evans, Dana Infante, Laura S. Craig

**Affiliations:** 1 Pacific Coastal and Marine Science Center, United States Geological Survey, Santa Cruz, California, United States of America; 2 Department of Geography, Dartmouth College, Hanover, New Hampshire, United States of America; 3 Forest and Rangeland Ecosystem Science Center, United States Geological Survey, Seattle, Washington, United States of America; 4 Cascades Volcano Observatory, Volcano Science Center, United States Geological Survey, Vancouver, Washington, United States of America; 5 Oregon Water Science Center, United States Geological Survey Portland, Oregon, United States of America; 6 Columbia River Research Laboratory, Western Fisheries Research Center, United States Geological Survey, Cook, Washington, United States of America; 7 Denver Federal Center, United States Geological Survey, Lakewood, Colorado United States of America; 8 Fort Collins Science Center, United States Geological Survey, Fort Collins, Colorado, United States of America; 9 Department of Geology, Bowling Green State University, Bowling Green, Ohio, United States of America; 10 Department of Fisheries and Wildlife, Michigan State University, East Lansing, Michigan, United States of America; 11 American Rivers, Washington, D.C., United States of America; University of Hyogo, JAPAN

## Abstract

Dams have been a fundamental part of the U.S. national agenda over the past two hundred years. Recently, however, dam removal has emerged as a strategy for addressing aging, obsolete infrastructure and more than 1,100 dams have been removed since the 1970s. However, only 130 of these removals had any ecological or geomorphic assessments, and fewer than half of those included before- and after-removal (BAR) studies. In addition, this growing, but limited collection of dam-removal studies is limited to distinct landscape settings. We conducted a meta-analysis to compare the landscape context of existing and removed dams and assessed the biophysical responses to dam removal for 63 BAR studies. The highest concentration of removed dams was in the Northeast and Upper Midwest, and most have been removed from 3^rd^ and 4^th^ order streams, in low-elevation (< 500 m) and low-slope (< 5%) watersheds that have small to moderate upstream watershed areas (10–1000 km^2^) with a low risk of habitat degradation. Many of the BAR-studied removals also have these characteristics, suggesting that our understanding of responses to dam removals is based on a limited range of landscape settings, which limits predictive capacity in other environmental settings. Biophysical responses to dam removal varied by landscape cluster, indicating that landscape features are likely to affect biophysical responses to dam removal. However, biophysical data were not equally distributed across variables or clusters, making it difficult to determine which landscape features have the strongest effect on dam-removal response. To address the inconsistencies across dam-removal studies, we provide suggestions for prioritizing and standardizing data collection associated with dam removal activities.

## Introduction

Dams have been a fundamental part of the U.S. national agenda and economic-development ideology over the past two hundred years because of their essential role in flood control, municipal water supply, power generation, and irrigation. In the past several decades, however, there has been a paradigm shift in dam and watershed management—driven by environmental, economic, and engineering concerns—leading to the removal of obsolete, unsafe, and economically non-viable dams emerging as a significant management and restoration strategy. This new agenda has led to the removal of > 1,000 dams in the past few decades [1, 2], yet scientific assessment of the effects of dam removal lags the rate of removal [2], a theme typical of other river restoration efforts nationally [3–5]. Though the scientific community has been studying various aspects of dam removal in limited capacity for the last few decades, there is still a need to provide resource managers with basic information about the likely effects of dam removal that could affect the cost, planning process, permit requirements, and monitoring components of dam removal projects. Moreover, dam removal studies have not been conducted across a broad enough range of landscapes to establish a predictive framework linking the context of the dam location to anticipated outcomes affecting river hydrology, channel morphology, sediment budgets, water quality, and ecological trajectories.

General lessons regarding river response to dam removal, however, are slowly emerging. These lessons can help identify fundamental operative processes and biophysical responses to dam removal and further enlighten management decisions [6–8]. Multiple factors drive the variability in geomorphic responses to dam removal, including dam size; removal method; reservoir size and shape; sediment volume, cohesiveness, and grain size; and released sediment volume relative to background sediment flux [1, 6, 9, 10]. Contrary to some perceptions, Major et al. [11] and East et al. [12] found that river channels can stabilize relatively quickly after dam removal—within months or years, not decades—approaching pre-dam emplacement morphology.

Ecological response trajectories after dam removal are difficult to generalize because response rates can be highly variable across taxa [13] and can be affected by past and current conditions [14–16]. In addition, most dam removal studies are short in duration and focus on a single response metric [2]. Despite these limitations, some patterns have emerged from the literature: there may be a lag between geomorphic and ecological responses [17, but see 18]; aquatic species typical of flowing rivers (lotic habitats) tend to replace stillwater (lentic) communities in the reservoir after dam removal [15]; and upstream fish migration that was formerly impeded by the dam may occur swiftly after dam removal in some cases [19–22].

Biophysical river responses to dam removal are affected by the surrounding landscape, but these effects are poorly understood [15, 23] because the literature consists mainly of specific case studies focused on short-term responses with limited comparison across regions. Without understanding a site’s landscape context (i.e., location within a watershed or regional and local patterns of climate, geology, and vegetation), it is difficult to interpret the broad applicability or local limitations of the biophysical responses to dam removal [24]. As a result, our fundamental understanding of long-term trajectories and broad-scale patterns of ecological, geomorphic, and hydrologic responses to dam removal is lacking. Furthermore, the breadth (or lack thereof) of published studies is directly tied to the expertise of researchers in each case study; truly interdisciplinary studies are rarely conducted for the same dam removal [2], and studies that integrate the trajectories of physical and biological responses are even more rare [25, but see 26].

To assess the state of science for understanding outcomes to dam removal and to determine the representativeness of dam removal case studies compared to the national dam population, we conducted a meta-analysis examining the landscape context—including natural and anthropogenic factors—of more than 50,000 existing dams and nearly 900 removed dams in the conterminous U.S. We also reviewed 104 published studies [27] with before- and after-removal (BAR) data from 63 dam removals to analyze the influence of landscape context in driving the biophysical response to dam removal. Because removals have occurred in settings where the human footprint may influence the response trajectory, we characterized landscape context as a combination of natural (e.g., ecoregion, watershed size) and anthropogenic (e.g., population density, transportation infrastructure) attributes. We used this approach to assess the landscape context of dams and removed dams; examine the biophysical response of river systems in different landscape settings; and highlight landscape contexts where additional research is needed. In doing so, we attempt to unpack “environmental context” into more specifically defined statistical associations but with the full knowledge that we are working with limited and geographically biased data. Finally, propose approaches for standardizing elements of dam-removal research that could increase our understanding of biophysical responses and help guide watershed management and restoration efforts. This type of comprehensive review has not been reported and our study is the first to formally examine the landscape context of dam removals with linked geospatial data at a national scale in the U.S. We also had access to a unique dam removal database compiled by American Rivers that allowed us to examine the geographic context for a larger population of dam removals than has been previously publicly available.

## Methods

We compiled geographic information for 50,772 existing dams listed in the National Anthropogenic Barrier Dataset (NABD) [28], a subset of dams from the 2009 National Inventory of Dams (U.S. Army Corps of Engineers– http://nid.usace.army.mil/cm_apex/f?p=838:12, accessed July 2010). We gathered the same information for the 874 removed dams included in the USGS Dam Removal Information Portal (DRIP– https://www.sciencebase.gov/drip/; accessed 1 July 2016). All existing and removed dams were linked to the National Hydrography Dataset Plus Version 1 (NHDPlusV1), allowing us to gather additional information from the National Fish Habitat Partnership’s (NFHP) 2015 National Assessment of Fish Habitat Condition Database [29, 30] and Anthropogenic Disturbance Database [31], as well as land-cover data summaries from the National Land Cover Database (NLCD– http://www.mrlc.gov/). From these sources, we identified natural and anthropogenic landscape-context factors in the river segment for each existing and removed dam (Table 1). We also examined the distribution of dams and dam removals in relation to Environmental Protection Agency (EPA) Level III Ecoregions, which characterize nation-wide landscape characteristics based on geology, landforms, soils, vegetation, climate, land use, wildlife, and hydrology [32] (https://www.epa.gov/eco-research/ecoregions; accessed 30 January 2017).

**Table 1:** Landscape variables.

Data obtained from the National Fish Habitat Partnership (version 2015)	
Data Type	Data description
*Catchment slope	Mean catchment slope (degrees)
*Catchment elevation	Mean catchment elevation (m)
*Groundwater index	Percent groundwater contribution to stream baseflow
*Precipitation	Mean annual precipitation (mm)
*Air temperature	Mean annual air temperature (C^o^)
*Habitat Condition Index	Index scoring the risk of habitat degradation for fish (scored as 0–5, with 0 representing very low risk of habitat degradation/very high fish habitat and 5 representing very high risk of habitat degradation/very poor fish habitat)
Population density	Census 2000 average population per catchment density (average population count/km^2^)
Road crossings	Road crossing density in the catchment (#/km^2^)
Toxic Release sites	Toxic Release Inventory (EPA) sites in the catchment (#/km^2^)
Superfund sites	EPA Superfund National Priority in the catchment (#/km^2^)
NPDES sites	National Pollutant Discharge Elimination System sites in the catchment (#/km^2^)
Water withdrawal	Total annual water withdrawal (million gallons per year–MGY)
Agriculture water withdrawal	Annual agriculture water withdrawal (MGY)
Domestic water withdrawal	Annual domestic water withdrawal (MGY)
Industrial water withdrawal	Annual industrial water withdrawal (MGY)
Thermoelectric water withdrawal	Annual thermoelectric water withdrawal (MGY)
Elevation at dam location	Elevation above sea level at the base of the dam location (m)
Data obtained from the National Land Cover Database (version 2006)	
Data Type	Data description
Open water	Percent of catchment
Perennial snow/ice	Percent of catchment
Developed open space	Percent of catchment
Developed low intensity	Percent of catchment
Developed medium intensity	Percent of catchment
Developed high intensity	Percent of catchment
Barren land	Percent of catchment
Deciduous forest	Percent of catchment
Evergreen forest	Percent of catchment
Mixed forest	Percent of catchment
Shrub/Scrub	Percent of catchment
Grassland/Herbaceous plants	Percent of catchment
Pasture/Hay	Percent of catchment
Cultivated crops	Percent of catchment
Woody wetlands	Percent of catchment
Emergent herbaceous wetlands	Percent of catchment

Landscape data obtained for existing and removed dams from the National Fish Habitat Partnership (NFHP) and the National Land Cover Database (NLCD). Landscape data were summarized within network catchments for the stream reaches immediately above the dams.

* indicates variables that were used in our analyses.

We generated summary statistics using these landscape characteristics for existing and removed dams to determine how representative removals have been of the overall dam population. We reduced the number of individual landscape factors used in our analyses because some of the variables were used to derive a habitat condition index (HCI) [29, 30], an index based on regionally specific responses of stream fishes to anthropogenic landscape factors. We excluded the variables used to create that index—including land cover, population density, road density, and the number of dams, mines, and point-source pollution sites—from subsequent analyses to avoid overrepresentation. The HCI uses a ranking of 1 through 5, with low scores corresponding to low risk of fish-habitat degradation and high scores to high risk of fish-habitat degradation.

We used seven variables—mean watershed slope, elevation, and area; precipitation; air temperature; ground water input; and HCI—in a Principal Components Analysis (PCA) to determine which landscape variables best explained the variability in landscape context among dam removals. Because landscape variables were measured using a variety of units, all variables were normalized prior to analysis (for each variable, the variable mean was subtracted from the value and then divided by the standard deviation). We also conducted a cluster analysis (using a resemblance matrix based on Euclidean distance) to determine whether dam removals formed significantly distinct clusters based on landscape context. We used similarity profile analysis (SIMPROF) to assign groupings for all dam removals and a subset of dam removals with BAR studies that had statistically different (*p* ≤ 0.01) landscape characteristics [33].

To determine if biophysical response to dam removal varied with landscape context, we selected a subset of dam removals from the DRIP database that had BAR data upstream of the reservoir, within the reservoir, and/or downstream of the dam site. When we accessed the database (November 2016), it contained information from 104 BAR studies from 63 dam removals (S1 Table). For each study, we classified the response to dam removal categorically for each biophysical variable as “increased,” “no change,” or “decreased.” We did not control for time frame of response (e.g., weeks to years after dam removal) following dam removal because the duration of studies was highly variable. For each variable (e.g., turbidity), we tallied the number of each response type, irrespective of methodological differences. We recognize that this way of analyzing the data resulted in a loss of resolution and somewhat limits our ability to compare across dam removals, but vagaries among studies required a level of generalization to assemble data coherently.

Based on the clusters determined in our SIMPROF analysis, we examined biophysical responses to dam removal within each geographic cluster having more than three dams and qualitatively compared the responses across geographic clusters. We could not conduct robust quantitative analyses on landscape context and biophysical responses because we were limited by the number of removals within each cluster, as well as by the overlap of data types among studies (S1 Table). We used PRIMER (v. 7, Primer Ltd.) and QGIS (v. 2.8.1) for all our analyses [34, 35].

## Results

**Fig 1:**
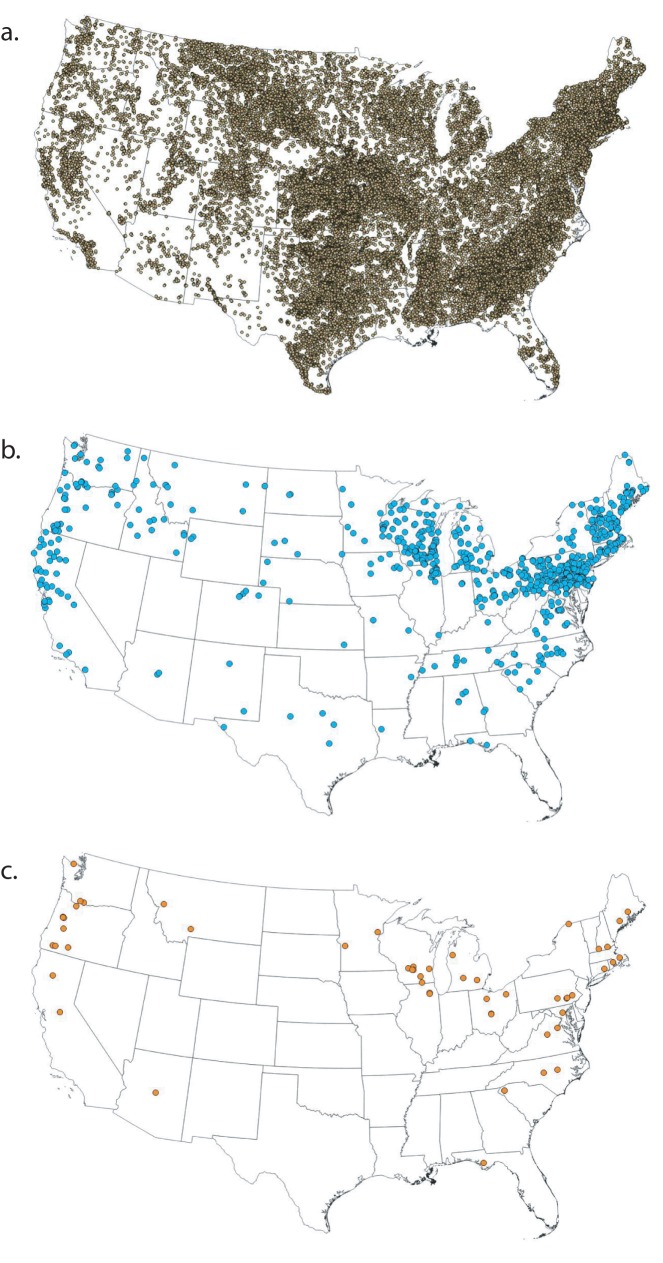
Geography of dams. U.S. distribution of (a) existing dams listed in the National Anthropogenic Barrier Dataset (*n* = 50,772); (b) removed dams from the Dam Removal Inventory Project (*n* = 874); (c) removed dams with before-after studies (n = 63).

**Fig 2:**
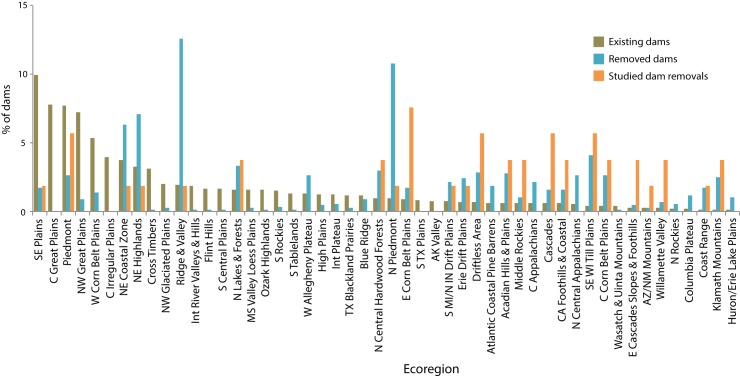
Ecoregions. EPA Level III Ecoregions for existing and removed dams in the U.S., and before-after-removal studies.

**Fig 3:**
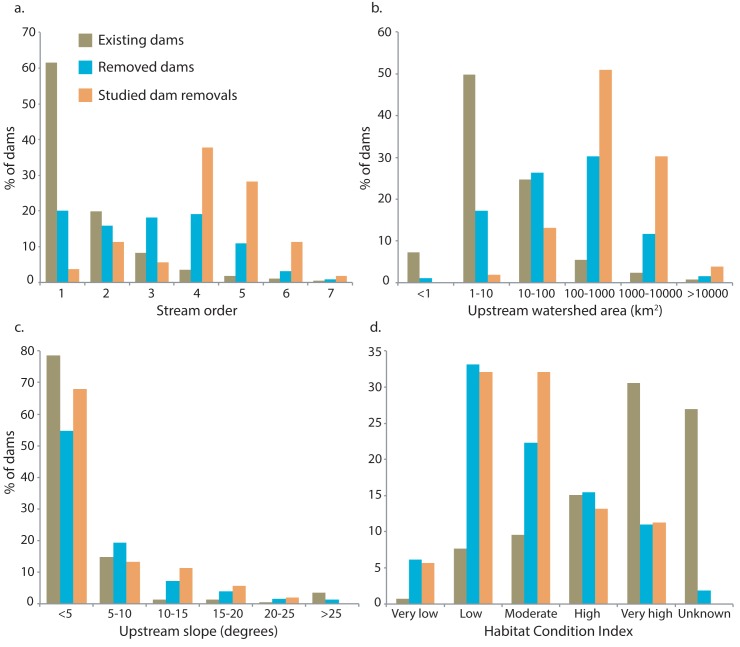
Landscape context. Comparison of (a) stream order; (b) watershed area (km^2^); (c) watershed slope (degrees); and (d) habitat condition index (risk of degradation) for existing (*n* = 50,772) and removed dams (*n* = 874), and dam removals with before- and after-removal studies (*n* = 63).

The densities of existing and removed dams, and studied dam removals varied greatly across the U.S. (Fig 1) [2]. The highest concentrations of existing dams were in the Southeastern Plains, Central Great Plains, Piedmont, and Northwestern Great Plains EPA Level III Ecoregions (Fig 2, S2 Table), predominantly on headwater streams (stream order = 1) that had small upstream watershed areas (< 10 km^2^) and low mean catchment slope (< 5 degrees) (Fig 3A–3C). Many were also located in areas where the HCI of the upstream catchment was very high, indicating an anthropogenic stressor(s) causes significant fish habitat degradation in those areas (Fig 3D). In contrast, the highest concentrations of dam removals have occurred in the Ridge and Valley, Northern Piedmont, Northeastern Highlands, and Northeastern Coastal Zone Ecoregions (Fig 2, S2 Table). Unlike existing dams, dam removals have occurred in a range of stream sizes, with a nearly equal number coming out of stream orders 1–4; and in river systems where the upstream catchment area is large, up to two orders of magnitude greater than that of existing dams (Fig 3B). Similar to existing dams, removed dams were located in watersheds with a low mean catchment slope (Fig 3C). Nearly 40% of removed dams were in watersheds with a low or very low risk of upstream habitat degradation (i.e., very low or low HCI score), and just over 30% of existing dams that were located in areas with a very high risk of habitat degradation (Fig 3D).

The landscape context of dam removals with BAR studies also differed in many respects from that of existing or removed dams (Figs 2 and 3). BAR studies were most numerous in the Eastern Corn Belt Plains (Ohio), Driftless Area (southern Wisconsin), Cascades (western U.S.), Southeastern Wisconsin Till Plains, and Piedmont Ecoregions (Fig 2, S2 Table). Except for the studies from the Eastern Corn Belt Plains and Cascades Ecoregions, these studies represented fewer than 13% of the removals in those areas (S2 Table). Studied dam removals have occurred predominantly on larger streams and in watersheds with low mean catchment slope and moderate to low risk of habitat degradation (HCI > 3) (Fig 3D).

**Fig 4:**
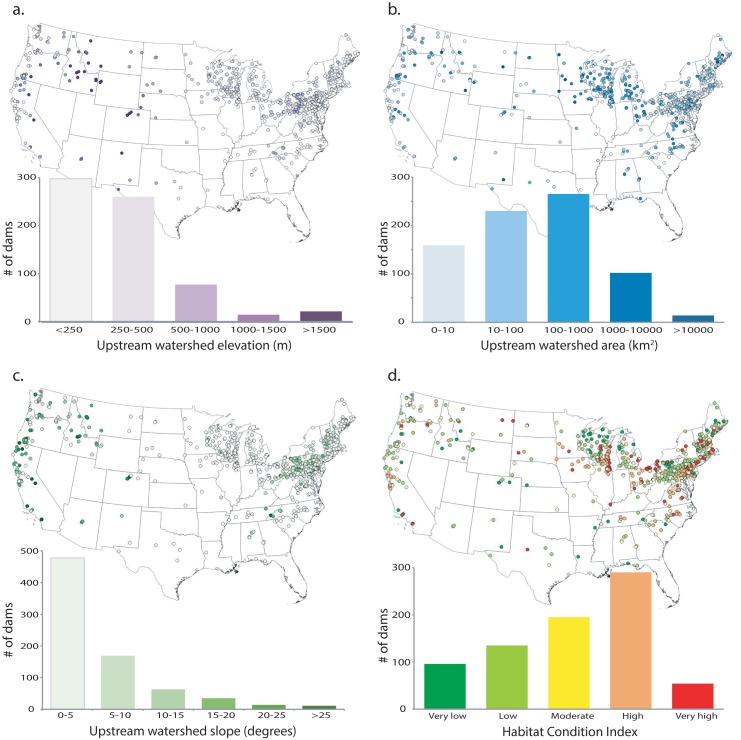
Spatial distribution of landscape characteristics. Spatial distribution of landscape characteristics for all removed dams: (a) upstream watershed elevation (m), (b) upstream watershed area (km^2^), (c) upstream watershed slope (degrees), and (d) habitat condition index (risk of habitat degradation).

We identified several notable spatial patterns in the landscape context of dam removals with respect to their distribution throughout the U.S. (Fig 4). The clusters of removals in the upper Midwest and upper New England generally occurred in large, low elevation, low slope watersheds, many with degraded fish habitat (Fig 4A–4D). In contrast, removals in the western U.S. were in high elevation, steep, small watersheds with predominantly moderate- to low-risk of habitat degradation (Fig 4A–4D). We identified a dearth of dam removals in central and south-central areas of the continental U.S., despite this area having one of the highest concentrations of existing dams (i.e., Central Great Plains).

**Fig 5:**
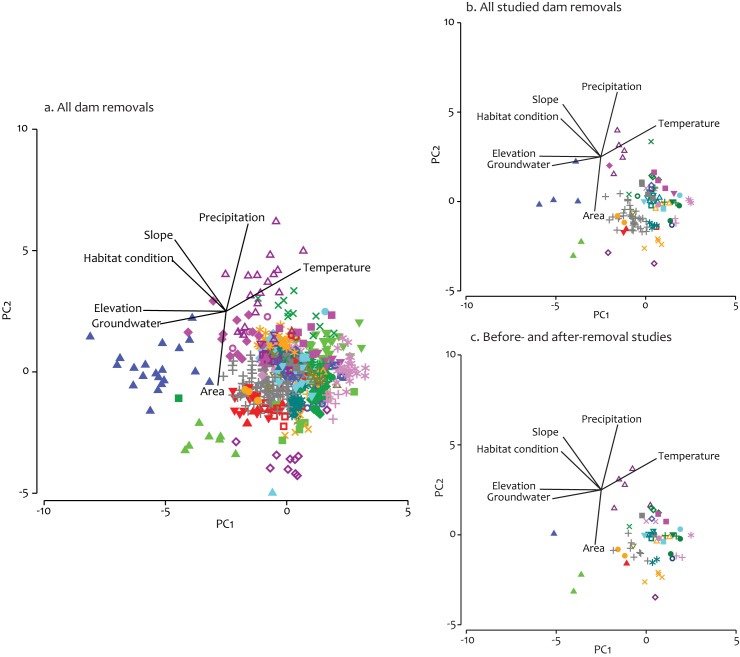
Principal Components Analysis results. Principal Components Analysis results for (a) all dam removals (57 clusters); (b) all studied dam removals (36 clusters); and (c) before-after studied dam removals (32 clusters). The number of clusters in (a) was determined from a cluster analysis; clusters in (b) and (c) show how many original clusters were represented in those subsets.

**Fig 6:**
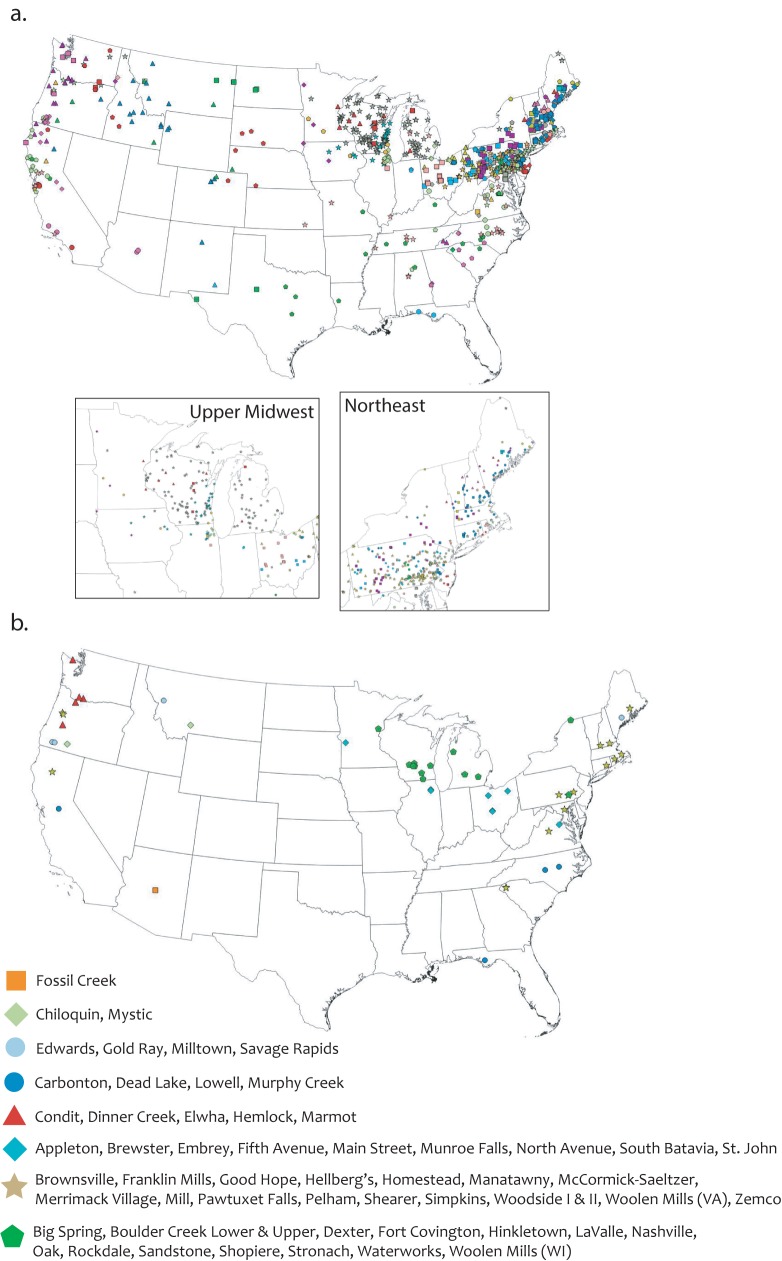
Spatial distribution of landscape clusters. (a) Spatial distribution of clusters based on landscape characteristics for all dam removals. Upper Midwest and Northeast sections magnified to show details. (b) Spatial distribution of clusters based on landscape characteristics of before-after studied dam removals.

For the seven variables we analyzed for all dam removals, the cluster analysis revealed 57 unique clusters of dam removals based on their landscape characteristics (Fig 5A). Although many of these clusters were concentrated in specific geographic regions, some removals that had similar landscape characteristics were widely distributed across the U.S. (Fig 6A). The PCA suggested the main factors differentiating the dam removal clusters were watershed elevation, groundwater input, and air temperature on principal component axis 1 (PC1); watershed area, watershed slope, and precipitation on PC2; and groundwater input, watershed elevation, and watershed slope on PC3 (Table 2); these three PCA axes explained 66% of the variation in landscape characteristics. Studied dam removals were represented in 36 of the 57 geographic clusters (Fig 5B); and BAR studies were conducted in 32 of the 57 geographic clusters (Fig 5C). Although over half of the geographic clusters had at least one BAR study, clusters in the lower right quadrant of the PCA axes had the greatest number of studies, representing large, low-elevation, and low-slope watersheds. Few BAR studies were conducted in small, high-elevation, high-slope watersheds (Fig 5C).

**Table 2:** Principal component loadings.

	PC 1	PC 2	PC 3	PC 4	PC 5
Eigenvalue	2.12	1.50	1.01	0.87	0.69
% Variation	30.3	21.4	14.4	12.5	9.9
Variables:					
Area	-0.55	-0.491	0.062	-0.787	-0.351
Slope	-0.339	0.473	0.461	-0.335	0.103
Elevation	-0.547	0.006	0.458	0.106	0.096
Groundwater	-0.436	-0.084	-0.568	-0.053	0.275
Temperature	0.493	0.280	0.218	-0.002	-0.275
Precipitation	0.146	0.579	-0.343	-0.484	0.260
Habitat condition	-0.358	0.340	-0.294	0.140	-0.799

Principal component loadings for the full PCA on all removed dams.

We conducted a separate cluster analysis based only on the landscape variables for BAR studies. This reduced analysis revealed eight significantly distinct clusters of removals, some with a wide geographic distribution (Fig 6B). Seven removals could not be categorized because data were not available for all landscape variables (Table 3). We used these new clusters to look at patterns of biophysical responses to dam removal.

**Table 3:** Landscape clusters for before and after-removal studies.

Cluster membership	Dam name and location
a (Mountain West)	Chiloquin, OR; Mystic, MT
b (West)	Edwards, ME; Gold Ray, OR; Milltown, MT; Savage Rapids, OR
c (Pacific Northwest)	Condit, WA; Dinner Creek, OR; Elwha, WA; Hemlock, WA; Marmot, OR
d (Arizona)	Fossil Creek, AZ
e (Upper Midwest)	Big Spring, WI; Boulder Creek (Lower & Upper), WI; Dexter, MI; Fort Covington, NY; Hinkletown, PA; LaValle, WI; Nashville, MI; Oak Street, WI; Rockdale, WI; Sandstone, MN; Shopiere, WI; Stronach, MI; Waterworks, WI; Woolen Mills, WI
f (New England)	Brownsville, OR; Franklin Mills, PA; Good Hope, PA; Hellberg’s, PA; Manatawny Creek, PA; McCormick-Saeltzer, CA; Merrimack Village, NH; Mill, NH; Pawtuxet Falls, RI; Shearer, OR; Simkins, MD; Sodom, OR; Woodside (I & II), SC; Woolen Mills, VA; Zemko, CT
g (Midwest)	Appleton, MN; Brewster Creek, IL; Embrey, VA; Fifth Avenue, OH; Main Street, OH; Munroe Falls, OH; North Avenue, WI; South Batavia, IL; St. John, OH
h (Southeast)	Carbonton, NC; Dead Lake, FL; Lowell, NC; Murphy Creek, CA
No cluster assigned due to missing landscape data	Central Avenue, OH; Homestead, NH; Off Billington Street, MA; Pelham, MA; Quaker Neck, NC; River Street, OH; Secor, OH

Significant clusters for before- and after-removal studies. Locations are indicated with abbreviations for states in the U.S. The cluster names in parentheses denote the region where a majority of the removals in each cluster were located.

**Fig 7:**
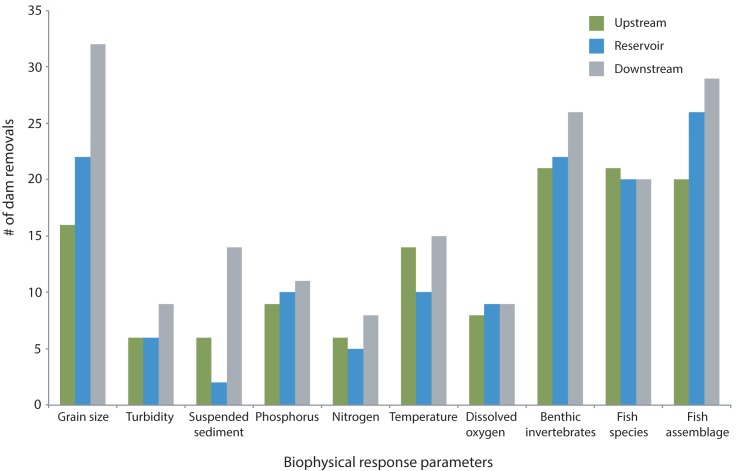
Before- and after-removal biophysical study parameters. Number of dam removals with upstream, reservoir, and downstream studies that reported the before- and after-removal responses of physical, water quality, and biological parameters.

Parameters reported most frequently across all BAR studies included sediment grain size, water temperature, aquatic invertebrates, and fish (Table 4). However, not all of these parameters were reported above the dam, within the reservoir, or downstream of the dam. For nearly all of the biophysical parameters we characterized, measurements were most frequently reported downstream of the removed dam (Fig 7). For example, approximately half of the BAR studies reported grain size measurements downstream of the former dam, while only 33% reported measurements within reservoir reaches and 25% in upstream reaches. Biotic variables were more consistently reported for all three river reaches than physical variables. Some variables were quantified using different metrics, particularly nutrients, aquatic invertebrates, and fish (Table 5).

**Table 4:** Reported biophysical metrics.

	Physical			Water quality				Biological		
Dam name and location (US state abbreviation)	Sediment grain size	Turbidity	Suspended-sediment concentration	Phosphate concentration	Nitrate concentration	Temperature	Dissolved oxygen	Aquatic invertebrates	Fish single species	Fish community
Appleton, MN										
Big Spring, WI										
Boulder Creek, WI*										
Brewster, IL										
Brownsville, OR										
Carbonton, NC										
Central Avenue, OH										
Chiloquin, OR										
Condit, WA										
Dead Lake, FL										
Dexter, MI										
Dinner Creek, OR										
Edwards, ME										
Elwha, WA										
Embrey, VA										
Fifth Avenue, OH										
Fort Covington, NY										
Fossil Creek, AZ										
Franklin Mills, PA										
Gold Ray, OR										
Good Hope, PA										
Hellberg’s, PA										
Hemlock, WA										
Hinkletown, PA										
Homestead, NH										
LaValle, WI										
Lowell, NC										
Main Street, OH										
Manatawny, PA										
Marmot, OR										
McCormick-Saeltzer, CA										
Merrimack Village, NH										
Mill, ME										
Milltown, MT										
Munroe Falls, OH										
Murphy Creek, CA										
Mystic, MT										
Nashville, MI										
North Avenue, WI										
Oak, WI										
Off Billington Street, MA										
Pawtuxet Falls, RI										
Pelham, MA										
Quaker Neck, NC										
River Street, OH										
Rockdale, WI										
Sandstone, MN										
Savage Rapids, OR										
Secor, OH										
Shearer, OR										
Shopiere, WI										
Simkins, MD										
Sodom, OR										
South Batavia, IL										
St. John, OH										
Stronach, MI										
Waterworks, WI										
Woodside, SC*										
Woolen Mills, VA										
Woolen Mills, WI										
Zemko, CT										
**Total:**	34	9	14	9	8	14	14	25	28	28

Biophysical metrics that were reported before and after dam removal; blank cell = no response reported, grey cell = response reported.

*In two instances, two dam removals were reported together in the literature–Upper and Lower Boulder Creek, WI, and Woodside I and II, SC.

**Table 5:** Measurement metrics.

*Phosphate*	*Nitrate*	*Aquatic Invertebrates*	*Target Fish*	*Fish Assemblage*
Total = 5	Total = 2	Abundance = 7	Abundance = 22	Abundance = 2
Dissolved = 4	Dissolved = 7	EPT abundance = 6	CPUE = 2	Biomass = 1
Particulate = 1	Particulate = 1	% EPT = 2	# of redds = 1	Composition = 7
SRP = 2		Diversity = 4	Size = 1	Diversity = 9
MRP = 1		Richness = 7		Richness = 6
		HBI score = 2		IBI = 2

Multiple metrics were used to measure the same parameter in before-after dam-removal studies. For each metric, the type of measurement reported is listed, followed by the number of dam removals using each metric. SRP–soluble reactive phosphorus; MRP–Molybdate reactive phosphorus; EPT–Ephemeroptera, Plecoptera, Trichoptera assemblage; HBI–Hilsenhoff biotic index; IBI–Index of biotic integrity.

**Fig 8:**
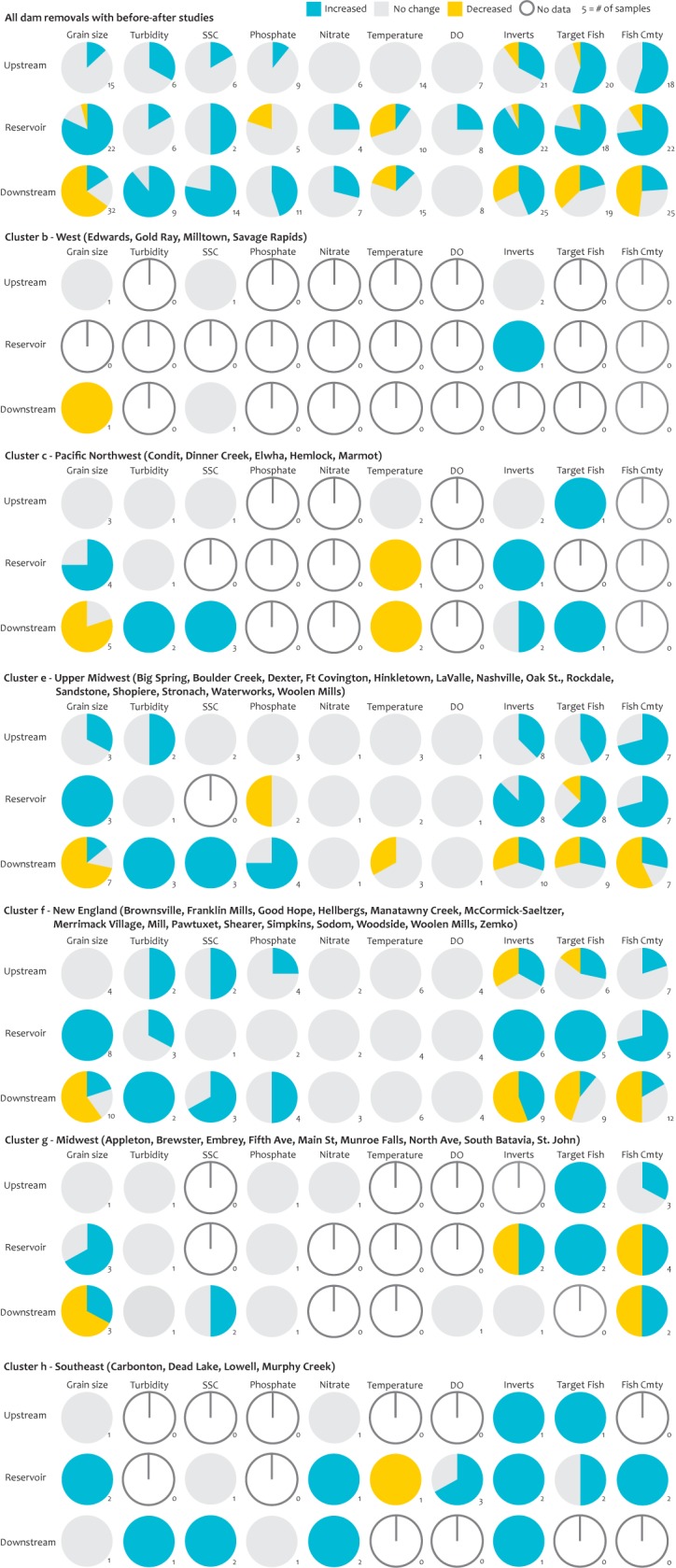
Biophysical responses to dam removal. Biophysical response for all before- and after-removal studies (top row) and within each distinct geographic cluster.

We could not formally test for differences in biophysical responses to dam removal because variables were not consistently reported across all dam removals in the eight geographic clusters (Fig 8). Water quality variables, including phosphate, nitrate, water temperature, and dissolved oxygen, were measured in only three of eight geographic clusters. In contrast, sediment grain size and fish species data were reported in all clusters (Fig 8). Physical responses to dam removal tended to be more consistent across geographic clusters than either water quality or ecological parameters. Sediment grain size tended to remain mostly unchanged in upstream reaches, coarsened in reservoir reaches, and fined downstream after dam removal; turbidity did not change upstream or in the reservoir reach but increased downstream; and suspended-sediment concentration increased downstream after dam removal. On the basis of limited measurements, water quality responses varied by geographic cluster, but many locations showed no change in water quality parameters in any of the three river reaches. In the Upper Midwest and New England clusters, however, phosphorus concentration (inclusive of all reported phosphate metrics listed in Table 5) increased downstream after dam removal, while nitrate concentration increased in the reservoir reach and downstream after dam removal in the Southeast cluster. Water temperature did not change in any river section after dam removal in a majority of studies, but after three removals—including two large removals in the Pacific Northwest—water temperature decreased downstream of the removed dams. A decrease in water temperature in the reservoir reach after dam removal was observed in only two of ten studies (Fig 8), yet a decrease in water temperature in the reservoir reach is assumed to be a typical response following dam removal [36].

Biological responses to dam removal were more variable than physical and water quality responses, particularly downstream of the dam. Aquatic invertebrate and fish (single species or community) responses varied among geographic clusters, and were also highly variable within a single geographic cluster (Fig 8). This variability was particularly evident at sites downstream of the dam in the two geographic clusters with the highest number of biological BAR studies, the Upper Midwest and New England, where there were nearly equal numbers of studies showing an increase, decrease, or no change in aquatic invertebrate and fish responses (Fig 8). In contrast, a majority of the BAR studies from reservoir and upstream reaches reported an increase in aquatic invertebrates or fish. BAR studies reporting the response of fish community composition were entirely absent from dam removals in the West and Pacific Northwest clusters—the clusters containing some of the largest dam removals.

## Discussion

Dam removals have occurred throughout the United States but have been concentrated in watersheds that represent a relatively narrow range of landscape characteristics compared to the characteristics of the existing dams throughout the U.S. Most dam removals have occurred in low-elevation watersheds with low catchment slope and large upstream areas, and most BAR studies were conducted in watersheds with similar characteristics. Watersheds in wet climates (high precipitation) with steep slopes, high mean elevations, and good fish habitat conditions (low chance of degradation) were poorly represented in BAR studies. Apparent geographic discrepancies between existing-dam density and removed dams may be due to factors related to economics, historical context, and dam function (e.g., irrigation, flood control, hydropower), but that information is rarely reported, and the discussion of those factors is beyond the scope of our analyses.

Biophysical responses to dam removal varied by geographic region, and not all biophysical variables were consistently reported after dam removals. Inconsistencies in the metrics reported, measurement timing, and study duration made it difficult to quantitatively assess biophysical responses in geographic regions with different landscape characteristics and predict how a system might respond to a dam removal based on its landscape context. We identified distinct differences in landscape context among existing and removed dams, and BAR studies. Our analysis comparing existing and removed dams, however, was limited to dams that were either ≥ 8 m tall with an 18,500-m^3^ or larger impoundment or ≥ 2 m tall with an impoundment at least 62,000 m^3^. Many removed dams did not meet those height or impoundment size requirements; of the 874 removed dams included in our analysis, only 165 of them met the criteria for being listed in the NID. Some states have more comprehensive inventories of existing dams, including small dams, but the NABD—which draws data from the NID—is the only publicly available list of existing dams throughout the country that is spatially linked to the NHDPlusV1.

Fish habitat condition index scores for existing dams were nearly the opposite of HCI scores for removed dams. Very low-quality fish habitat with high risk of degradation characterized many landscapes around existing dams, but dam removals have occurred in landscapes with moderate to high quality fish habitat with moderate to low risk of degradation. Dam removals may have been more common in areas with high quality fish habitat to enhance the probability of a successful outcome, particularly if ecosystem restoration was a goal of the dam removal. The HCI was calculated based on landscape characteristics of the watershed above a dam, and is an important metric to consider when planning dam removals because habitat quality within the watershed influences the biophysical responses to dam removal and the potential for habitat condition improvements [37]. River ecosystems may be more likely to recover to pre-dam conditions if dam emplacement is a main source of anthropogenic stressors on the landscape, contributing to an increased risk of habitat degradation (Table 6).

**Table 6:** Anthropogenic landscape context.

Cluster (HCI score)	Urban (%)	Forested (%)	Agriculture (%)	Population density (#/km^2^)	Road crossings (#/km^2^)	Water withdrawal (MGY)	Phosphorus input (kg/km/yr)	Nitrogen input (kg/km/yr)	Sediment input (kg/km/yr)
a–Mountain West (2.9 –moderate risk)	0.02	72.9	1.0	34.3	0.14	30.7	9.0	39.0	2292
b–West (2.1 –high risk)	1.5	63.0	5.2	9.3	0.31	16.6	16.7	58.0	6814
c–Pacific Northwest (2.9 –moderate risk)	1.0	82.1	0.8	10.1	0.17	3.6	7.1	68.1	17738
d–Arizona (3.3 –low risk)	0.1	60.3	0	5.2	0.17	3.6	2.1	8.5	4610
e–Upper Midwest (3.0 –moderate/low risk)	3.2	30.2	48.0	15.9	0.43	13.2	77.8	723	57149
f–New England (2.9 –moderate risk)	5.1	53.8	20.5	44.7	0.57	25.9	97.2	697	85414
g–Midwest (1.1 –high risk)	9.7	19.6	54.1	25.1	0.49	57.6	92.9	1288	71285
h–Southeast (3.0 –moderate/low risk)	2.6	30.7	20.4	77.9	0.44	108.4	49.0	321	51079

Anthropogenic landscape context for before- and after-removal studies clusters.

Watershed area, elevation, and precipitation were the dominant landscape variables separating the geographical clusters of removed dams. Of the 57 unique clusters identified in the cluster analysis, 43 were concentrated in the lower-right quadrant of the PC plot, representing large-area, low-elevation, and low-precipitation watersheds (Fig 5A). This pattern held for all studied dam removals (Fig 5B) and BAR studies (Fig 5C). This predominant clustering of removals suggests that our frame of reference for understanding the biophysical response to dam removal is quite limited. This was especially true for BAR studies, which rarely examined physical and biological responses.

For the eight clusters of dam removals with BAR data, we were unable to quantitatively compare the biophysical responses with respect to landscape context because reported variables were neither consistent nor standardized. With the exception of BAR studies in the New England cluster, each cluster had missing data for at least one of the ten metrics examined, and many variables were only reported in one dam removal in the cluster. For dam removals with landscape characteristics outside the main groupings, whole classes of response variables were missing, including water quality and fish response data (Fig 8).

A number of factors likely contributed to the variation in biophysical response among geographic clusters. Firstly, the metrics used to measure responses varied among dam removals. For example, in the papers we reviewed, investigators used five different metrics to measure changes in phosphorous concentration and six metrics to measure changes in aquatic invertebrates (Table 5). Secondly, metrics were reported over different temporal scales [2], thus potentially obscuring differences between short-term and long-term changes after dam removal. For instance, after the removal of the Boulder Creek dams in South Carolina, soluble reactive phosphorus concentration increased within hours [38], but concentrations decreased after two weeks. As a result, there was no significant long-term change in phosphate concentration before and after dam removal. Similarly, dissolved phosphate concentration increased when the Good Hope Mill Dam in Pennsylvania was breached, but it returned to pre-removal levels within hours [39]. Aquatic invertebrate and fish responses to dam removal, particularly downstream, were strongly dependent on study timing [40]. Studies conducted immediately after dam removal commonly showed a decrease in invertebrate and fish metrics downstream of a dam removal, especially if sediment grain size changed [41, 42]. In contrast, studies that were conducted after the initial pulse of sediment moved through the system following dam removal showed either no change or a positive effect of dam removal [43, 44]. Finally, the reported biological response to dam removal can be influenced by the species monitored. For instance, in our analysis, some aquatic invertebrate studies reported responses of species that were present before dam removal [45, 46] and others reported the responses of species that were expected to colonize after dam removal (i.e., EPT taxa) [47–49]. In the first case, species tended to decrease after dam removal, while in the latter case they tended to increase. Other studies reported species diversity or richness [40, 50–53], which can be difficult to interpret without species-specific information because those metrics may not change if an equal number of pre-removal species are replaced with post-removal species.

Some of the biophysical responses we found in the literature were unexpected. For instance, water temperature decreased in reservoir and downstream reaches in only 10% of studies we examined [47, 54, 55] and nutrients increased downstream in only 30% of studies [39, 45, 46, 48, 56–58]. We expected these percentages to be much higher based on assumptions of biophysical response to dam removal [15, 17, 59, 60]. Individual fish species and fish communities upstream of a dam removal did not change in 40% of dam removals examined, nor did they change in more than 25% of downstream sites examined. The scientific community needs more data to understand ecosystem response in order to inform management decisions and create realistic expectations for post-removal recovery.

Our analysis did not consider all of the possible variables that could contribute to biophysical response to dam removal, and we recognize that landscape context does not affect all responses. For example, at Fossil Creek Dam, Arizona, native fish density increased after flow was restored to the downstream system, but only in stream segments where invasive species had been removed [61].

To date, most dams have been removed for economic, safety, or liability reasons rather than to restore ecosystem function [62]. As with many restoration efforts, removal rationales are not always available, nor is there a singularly agreed upon reason for removing a dam. Therefore, the objectives (or lack thereof) for removing dams may affect the types of variables monitored before and after dam removal. However, every dam removal, no matter the rationale, is an opportunity to gain further insight into how ecosystems respond and how physical and biological responses are connected. Recognizing the need for studies to remain focused on their objectives, we suggest that variables sampled before and after dam removal be prioritized and protocols developed in an attempt to coordinate and standardize the type of data that are collected. Many studies on dam removals were not comparable because of differences in metrics measured, methodologies employed, and study interval, including whether or not pre- and post-removal data were collected. Standardization of the type, frequency, and duration of data collection can help the scientific community better understand how the responses of river ecosystems vary as a function of landscape context. The following approaches provide examples of potential standardized procedures for evaluating biophysical responses of river systems to dam removal:

1. Sample before and after dam removal and at temporal and spatial scales that are meaningful for the metrics sampled and the magnitude of anticipated change.
2. Sample upstream, within the reservoir, and downstream of the proposed dam removal site. Studies that focus solely on the downstream response to dam removal do not provide a comprehensive view of biophysical response.
3. Sample a broad range of metrics for comparative purposes. If that is not possible, prioritize response measurements for indicator species that have known relationships to other variables.
4. Use technology and citizen science to expand the duration of the sampling or monitoring program. Satellite images are becoming increasingly available (e.g., Digital Globe) and can be used to assess landform and vegetation changes. Enlist citizen scientists to take spatially aligned repeat photographs, measure stream temperature, or record other parameters from fixed locations.
5. Compile, preserve and publically release data. Add data to public databases, including the Dam Removal Information Portal (https://www.sciencebase.gov/drip/).

## Conclusion

Dam removal has become an increasingly common restoration strategy, and new efforts are underway to help prioritize removals and guide removal decision-making [63]. Management decisions to remove dams are beginning to adopt a range of strategies, from a “hot spot” approach [64] to more overt economic strategies for prioritizing barrier removal [65, 66], to those aimed at achieving broader ecological gains [67, 68]. Our results indicate that landscape context may inform possible biophysical responses to removal, but a broader geographic range of removals is required. Thus, along with other management priorities, decisions about dam removal might consider where the proposed removal is located and how its removal can help advance our understanding of biophysical responses of river systems. Dam removals are large-scale experiments that offer tremendous opportunities to understand fluvial systems and the influence of humans on watershed processes and ecosystem dynamics. Knowledge of biophysical responses to dam removal in a regional context can be leveraged to anticipate the effects of pending dam removals and to help coordinate management efforts to meet conservation and restoration goals.

## Supporting information

S1 Table Before- and after-removal studies. Before-after-removal studies used in our statistical analysis; biophysical parameters measured in each study are indicated with grey shading.
(DOCX)

S2 Table EPA Level I, II, and III Ecoregions. EPA Level I, II, and III Ecoregion classifications (https://www.epa.gov/eco-research/ecoregions-north-america) for dams listed in the National Anthropogenic Barrier Dataset (NABD), removed dams in the USGS Dam Removal Information Portal (DRIP), and removed dams with before- and after-removal studies (BAR).
(DOCX)
