# Supplementary material for: Landscape context and the biophysical response of rivers to dam removal in the United States
Source: PLoS One. 2017 Jul 10;12(7):e0180107. doi: 10.1371/journal.pone.0180107 (PMC5503210; doi:10.1371/journal.pone.0180107)
Supplement: S1 Table — Before-after-removal studies used in our statistical analysis; biophysical parameters measured in each study are indicated with grey shading. (DOCX) [file pone.0180107.s001.docx]

Supporting information Table 1. Before-after-removal studies and the biophysical parameters measured in each study indicated with grey shading.

| Citation | Dam name | State | Grain size | Turbidity | Suspended sediment concentration | Phosphorus concentration | Nitrate concentration | Temperature | Dissolved oxygen | Aquatic invertebrates | Fish single species | Fish community |
| --- | --- | --- | --- | --- | --- | --- | --- | --- | --- | --- | --- | --- |
| Katapodis and Aadland [1] | Appleton | MN |  |  |  |  |  |  |  |  |  |  |
| Kornis et al. [2] | Big Spring | WI |  |  |  |  |  |  |  |  |  |  |
| Orr et al. [3] | Boulder Creek | WI |  |  |  |  |  |  |  |  |  |  |
| Orr et al. [4] | Boulder Creek | WI |  |  |  |  |  |  |  |  |  |  |
| Rogers [5] | Boulder Creek | WI |  |  |  |  |  |  |  |  |  |  |
| Stanley et al. [6] | Boulder Creek | WI |  |  |  |  |  |  |  |  |  |  |
| Kosky et al. [7] | Brewster | IL |  |  |  |  |  |  |  |  |  |  |
| Straub [8] | Brewster | IL |  |  |  |  |  |  |  |  |  |  |
| Tullos et al. [9] | Brownsville | OR |  |  |  |  |  |  |  |  |  |  |
| Zunka [10] | Brownsville | OR |  |  |  |  |  |  |  |  |  |  |
| Kibler et al. [11] | Brownsville | OR |  |  |  |  |  |  |  |  |  |  |
| Heise et al. [12] | Carbonton | NC |  |  |  |  |  |  |  |  |  |  |
| Howard et al. [13] | Carbonton | NC |  |  |  |  |  |  |  |  |  |  |
| DeShon [14] | Central Ave | OH |  |  |  |  |  |  |  |  |  |  |
| Martin et al. [15] | Chiloquin | OR |  |  |  |  |  |  |  |  |  |  |
| Colaiacomo [16] | Condit | WA |  |  |  |  |  |  |  |  |  |  |
| Engle et al. [17] | Condit | WA |  |  |  |  |  |  |  |  |  |  |
| Hatten et al. [18] | Condit | WA |  |  |  |  |  |  |  |  |  |  |
| Jolley et al. [19] | Condit | WA |  |  |  |  |  |  |  |  |  |  |
| Wilcox et al. [20] | Condit | WA |  |  |  |  |  |  |  |  |  |  |
| Hill et al. [21] | Dead Lake | FL |  |  |  |  |  |  |  |  |  |  |
| Knittel [22] | Dexter | MI |  |  |  |  |  |  |  |  |  |  |
| Stewart [23] | Dinner Creek | OR |  |  |  |  |  |  |  |  |  |  |
| Casper et al. [24] | Edwards | ME |  |  |  |  |  |  |  |  |  |  |
| East et al. [25] | Elwha | WA |  |  |  |  |  |  |  |  |  |  |
| Hitt et al. [26] | Embrey | VA |  |  |  |  |  |  |  |  |  |  |
| Dorobek et al. [27] | Fifth Ave. | OH |  |  |  |  |  |  |  |  |  |  |
| Cooper [28] | Fort Covington | NY |  |  |  |  |  |  |  |  |  |  |
| Cooper [29] | Fort Covington | NY |  |  |  |  |  |  |  |  |  |  |
| Compson et al. [30] | Fossil Creek | AZ |  |  |  |  |  |  |  |  |  |  |
| Dinger [31] | Fossil Creek | AZ |  |  |  |  |  |  |  |  |  |  |
| Marks et al. [32] | Fossil Creek | AZ |  |  |  |  |  |  |  |  |  |  |
| Muehlbauer et al. [33] | Fossil Creek | AZ |  |  |  |  |  |  |  |  |  |  |
| Hutchison [34] | Franklin Mills | PA |  |  |  |  |  |  |  |  |  |  |
| Stephens [35] | Gold Ray Dam | OR |  |  |  |  |  |  |  |  |  |  |
| Chaplin et al. [36] | Good Hope | PA |  |  |  |  |  |  |  |  |  |  |
| Hutchison [34] | Hellberg | PA |  |  |  |  |  |  |  |  |  |  |
| Claeson & Coffin [37] | Hemlock | WA |  |  |  |  |  |  |  |  |  |  |
| Hutchison [34] | Hinkletown | PA |  |  |  |  |  |  |  |  |  |  |
| Gartner et al. [38] | Homestead | NH |  |  |  |  |  |  |  |  |  |  |
| Catalano [39] | LaValle | WI |  |  |  |  |  |  |  |  |  |  |
| Catalano et al. [40] | LaValle | WI |  |  |  |  |  |  |  |  |  |  |
| Doyle et al. [41] | LaValle | WI |  |  |  |  |  |  |  |  |  |  |
| Riggsbee et al. [42] | Lowell | NC |  |  |  |  |  |  |  |  |  |  |
| Sherman [43] | Lowell | NC |  |  |  |  |  |  |  |  |  |  |
| Dorobek et al. [27] | Main St. | OH |  |  |  |  |  |  |  |  |  |  |
| Bushaw‐Newton et al. [44] | Manatawny | PA |  |  |  |  |  |  |  |  |  |  |
| Thomson et al. [45] | Manatawny | PA |  |  |  |  |  |  |  |  |  |  |
| Velinsky et al. [46] | Manatawny | PA |  |  |  |  |  |  |  |  |  |  |
| Zunka [10] | Marmot | OR |  |  |  |  |  |  |  |  |  |  |
| Cui et al. [47] | Marmot | OR |  |  |  |  |  |  |  |  |  |  |
| Keith [48] | Marmot | OR |  |  |  |  |  |  |  |  |  |  |
| Podolak [49] | Marmot | OR |  |  |  |  |  |  |  |  |  |  |
| Ferry & Miller [50] | McCormick-Saeltzer | CA |  |  |  |  |  |  |  |  |  |  |
| Greenwald et al. [51] | McCormick-Saeltzer | CA |  |  |  |  |  |  |  |  |  |  |
| Simons et al. [52] | McCormick-Saeltzer | CA |  |  |  |  |  |  |  |  |  |  |
| Conlon [53] | Merrimack Village | NH |  |  |  |  |  |  |  |  |  |  |
| Pearson et al. [54] | Merrimack Village | NH |  |  |  |  |  |  |  |  |  |  |
| Hogg et al. [55] | Mill | ME |  |  |  |  |  |  |  |  |  |  |
| Lambing & Sando [56] | Milltown | MT |  |  |  |  |  |  |  |  |  |  |
| Sando & Lambing [57] | Milltown | MT |  |  |  |  |  |  |  |  |  |  |
| Peck & Kasper [58] | Munroe Falls | OH |  |  |  |  |  |  |  |  |  |  |
| Rumschlag & Peck [59] | Munroe Falls | OH |  |  |  |  |  |  |  |  |  |  |
| Ahearn & Dahlgren [60] | Murphy Creek | CA |  |  |  |  |  |  |  |  |  |  |
| Schmitz et al. [61] | Mystic | MT |  |  |  |  |  |  |  |  |  |  |
| Gibson et al. [62] | Nashville | MI |  |  |  |  |  |  |  |  |  |  |
| Hirethota et al. [63] | North Avenue | WI |  |  |  |  |  |  |  |  |  |  |
| Catalano et al. [40] | Oak | WI |  |  |  |  |  |  |  |  |  |  |
| Orr & Koenig [4] | Oak | WI |  |  |  |  |  |  |  |  |  |  |
| Stanley et al. [64] | Oak | WI |  |  |  |  |  |  |  |  |  |  |
| Conlon [65] | Off Billington St. | MA |  |  |  |  |  |  |  |  |  |  |
| Cantwell et al. [66] | Pawtuxet Falls | RI |  |  |  |  |  |  |  |  |  |  |
| Magilligan et al. [67] | Pelham | MA |  |  |  |  |  |  |  |  |  |  |
| Bowman [68] | Quaker Neck | NC |  |  |  |  |  |  |  |  |  |  |
| Burdick & Hightower [69] | Quaker Neck | NC |  |  |  |  |  |  |  |  |  |  |
| DeShon [14] | River St | OH |  |  |  |  |  |  |  |  |  |  |
| Doyle et al. [41] | Rockdale | WI |  |  |  |  |  |  |  |  |  |  |
| Katopodis et al. [1] | Sandstone | MN |  |  |  |  |  |  |  |  |  |  |
| Tullos et al. [9] | Savage Rapids | OR |  |  |  |  |  |  |  |  |  |  |
| Evans et al. [70] | Secor | OH |  |  |  |  |  |  |  |  |  |  |
| Gottgens [71] | Secor | OH |  |  |  |  |  |  |  |  |  |  |
| Harris [72] | Secor | OH |  |  |  |  |  |  |  |  |  |  |
| Tullos et al. [73] | Shearer | OR |  |  |  |  |  |  |  |  |  |  |
| Pollard & Reed [74] | Shopiere | WI |  |  |  |  |  |  |  |  |  |  |
| Harbold et al. [75] | Simkins | MD |  |  |  |  |  |  |  |  |  |  |
| Tullos et al. [73] | Sodom | OR |  |  |  |  |  |  |  |  |  |  |
| Maloney et al. [76] | South Batavia | IL |  |  |  |  |  |  |  |  |  |  |
| Cheng & Granata [77] | St. John | OH |  |  |  |  |  |  |  |  |  |  |
| Granata et al. [78] | St. John | OH |  |  |  |  |  |  |  |  |  |  |
| Krieger & Stearns [79] | St. John | OH |  |  |  |  |  |  |  |  |  |  |
| Nechvatal [80] | St. John | OH |  |  |  |  |  |  |  |  |  |  |
| Burroughs [81] | Stronach | MI |  |  |  |  |  |  |  |  |  |  |
| Burroughs [82] | Stronach | MI |  |  |  |  |  |  |  |  |  |  |
| Burroughs et al. [83] | Stronach | MI |  |  |  |  |  |  |  |  |  |  |
| Mistak [84] | Stronach | MI |  |  |  |  |  |  |  |  |  |  |
| Catalano [39] | Waterworks | WI |  |  |  |  |  |  |  |  |  |  |
| Catalano et al. [40] | Waterworks | WI |  |  |  |  |  |  |  |  |  |  |
| Stanley et al. [85] | Waterworks | WI |  |  |  |  |  |  |  |  |  |  |
| Bulak et al. [86] | Woodside | SC |  |  |  |  |  |  |  |  |  |  |
| Marion [87] | Woodside | SC |  |  |  |  |  |  |  |  |  |  |
| Whitener [88] | Woodside | SC |  |  |  |  |  |  |  |  |  |  |
| Doucette [89] | Woolen Mills | VA |  |  |  |  |  |  |  |  |  |  |
| Kanehl et al. [90] | Woolen Mills | WI |  |  |  |  |  |  |  |  |  |  |
| Poulos et al. [91] | Zemko | CT |  |  |  |  |  |  |  |  |  |  |

1. Katopodis C, Aadland L. Effective dam removal and river channel restoration approaches. International Journal of River Basin Management. 2006;4(3):153-68.

2. Kornis MS, Weidel BC, Powers SM, Diebel MW, Cline TJ, Fox JM, et al. Fish community dynamics following dam removal in a fragmented agricultural stream. Aquatic Sciences. 2015;77(3):465-80.

3. Orr CH, Kroiss SJ, Rogers KL, Stanley EH. Downstream benthic responses to small dam removal in a coldwater stream. River Research and Applications. 2008;24(6):804-22.

4. Orr CH, Rogers KL, Stanley EH. Channel morphology and P uptake following removal of a small dam. Journal of the North American Benthological Society. 2006;25(3):556-68.

5. Rogers KL. Temporary downstream benthic responses to small-dam removal: University of Wisconsin--Madison; 2006.

6. Stanley EH, Catalano MJ, Mercado-Silva N, Orr C. Short communication: effects of dam removal on brook trout in a Wisconsin stream. River Research and Applications. 2007;23:792-98.

7. Kosky KM, Straub TD, Roseboom DP, Johnson GP. Preliminary results of a dam-removal analysis on Brewster Creek near St. Charles, Illinois, 2002–2004. Proceedings of the 2004 Self-Sustaining Solutions for Streams, Wetlands, and Watersheds Conference; 2004; St. Paul, Minnesota2004. p. 266-72.

8. Straub T. Erosion dynamics of a stepwise small dam removal, Brewster Creek Dam near St. Charles, Illinois: Colorado State University; 2007.

9. Tullos DD, Finn DS, Walter C. Geomorphic and ecological disturbance and recovery from two small dams and their removal. PLoS One. 2014;9(9):e108091.

10. Zunka JPP. Dam removals and downstream bar-pool morphology: Oregon State University; 2011.

11. Kibler K, Tullos D, Kondolf M. Evolving expectations of dam removal outcomes: Downstream geomorphic effects following removal of a small, gravel‐filled Dam1. JAWRA Journal of the American Water Resources Association. 2011;47(2):408-23.

12. Heise RJ, Cope WG, Kwak TJ, Eads CB. Short-term effects of small dam removal on a freshwater mussel assemblage. WALKERANA The Journal of the Freshwater Mollusk Conservation Society. 2013;16(1):41-52.

13. Howard G, Cusack M, Savidge T. Carbonton Dam - Deep River Watershed restoration site. Ecosystem Enhancement Program: 2007.

14. DeShon J. Biological and habitat study of the Olentangy River, 2005 and 2009. Ohio Environmental Protection Agency, Technical Report EAS/2010-5-8: 2010.

15. Martin BA, Hewitt DA, Ellsworth CM. Effects of Chiloquin Dam on spawning distribution and larval emigration of Lost River, shortnose, and Klamath largescale suckers in the Williamson and Sprague Rivers, Oregon. U.S. Geological Survey, Open-File Report 2013-1039: 2013.

16. Colaiacomo EJ. Downstream spatial and temporal response to dam removal, White Salmon River, WA: University of Montana; 2014.

17. Engle R, Skalicky J, Poirier J. Translocation of Lower Columbia River Fall Chinook Salmon (Oncorhynchus tshawytscha) in the year of Condit Dam removal and year one post-removal assessments. 2011 and 2012 Report. U.S. Fish and Wildlife Service, Columbia River Fisheries Program Office: 2012.

18. Hatten JR, Batt TR, Skalicky JJ, Engle R, Barton GJ, Fosness RL, et al. Effects of dam removal on Tule fall Chinook salmon spawning habitat in the White Salmon River, Washington. River Research and Applications. 2015.

19. Jolley JC, Silver GS, Whitesel TA. Occurrence, detection, and habitat use of larval Lamprey in the Lower White Salmon River and mouth: Post-Condit Dam removal. FY 2012 Annual Report. U.S. Fish and Wildlife Service: 2013.

20. Wilcox AC, O'Connor JE, Major JJ. Rapid reservoir erosion, hyperconcentrated flow, and downstream deposition triggered by breaching of 38 m tall Condit Dam, White Salmon River, Washington. Journal of Geophysical Research: Earth Surface. 2014;119(6):1376-94.

21. Hill MJ, Long EA, Hardin S. Effects of dam removal on Dead Lake, Chipola River, Florida. Southeast Association of Fish and Wildlife Agencies1994. p. 512-23.

22. Knittel MJ. The impact of a small dam removal on benthic macroinvertebrate Ccommunities of Mill Creek (Washtenaw Co., MI.): University of Michigan; 2010.

23. Stewart GB. Patterns and processes of sediment transport following sediment-filled dam removal in gravel bed rivers: Oregon State University; 2006.

24. Casper AF, Thorp JH, Davies SP, Courtemanch DL. Ecological responses of zoobenthos to darn removal on the Kennebec River, Maine, USA. Archiv Hydrobiologie, Supplement. 2006;158(4):541-55.

25. East AE, Pess GR, Bountry JA, Magirl CS, Ritchie AC, Logan JB, et al. Large-scale dam removal on the Elwha River, Washington, USA: River channel and floodplain geomorphic change. Geomorphology. 2015;228:765-86. doi: 10.1016/j.geomorph.2014.08.028.

26. Hitt NP, Eyler S, Wofford JE. Dam removal increases American eel abundance in distant headwater streams. Transactions of the American Fisheries Society. 2012;141(5):1171-9.

27. Dorobek A, Sullivan SMP, Kautza A. Short-term consequences of lowhead dam removal for fish assemblages in an urban river system. River Systems. 2015;21(2-3):125-39.

28. Cooper JE. Unionid mussel mortality from habitat loss in the Salmon River, New York, following dam removal. In: Daniels JA, editor. Advances in Environmental Research. 142011. p. 351-64.

29. Cooper JE. Effect of Dam Removal on Aquatic Communities in the Salmon River, New York Cooper Environmental Research: 2013.

30. Compson ZG, Mier MZ, Marks JC. Effects of travertine and flow on leaf retention in Fossil Creek, Arizona. Hydrobiologia. 2009;630(1):187-97.

31. Dinger EC. Aquatic conservation biology in arid ecosystems: Northern Arizona University; 2006.

32. Marks JC, Haden GA, O’Neill M, Pace C. Effects of flow restoration and exotic species removal on recovery of native fish: lessons from a dam decommissioning. Restoration Ecology. 2010;18(6):934-43.

33. Muehlbauer JD, LeRoy CJ, Lovett JM, Flaccus KK, Vlieg JK, Marks JC. Short-term responses of decomposers to flow restoration in Fossil Creek, Arizona, USA. Hydrobiologia. 2009;618(1):35-45.

34. Hutchison B. Fish and macroinvertebrate assemblages following the removal of low-head dams in two Pennsylvania streams: Pennsylvania State University; 2008.

35. Stephens JL. Short term response of vegetation and birds to dam removal on the Rogue River, Oregon. Klamath Bird Observatory, Report Number KBO-2014-0011, 2014.

36. Chaplin JJ, Brightbill RA, Bilger MD. Effects of removing Good Hope Mill Dam on selected physical, chemical, and biological characteristics of Conodoguinet Creek, Cumberland County, Pennsylvania. Report. U.S. Geological Survey Scientific Investigations Report 2005-5226: 2005 2005-5226.

37. Claeson S, Coffin B. Physical and Biological Responses to an Alternative Removal Strategy of a Moderate‐sized Dam in Washington, USA. River Research and Applications. 2015.

38. Gartner JD, Magilligan FJ, Renshaw CE. Predicting the type, location and magnitude of geomorphic responses to dam removal: Role of hydrologic and geomorphic constraints. Geomorphology. 2015;251:20-30.

39. Catalano MJ. Evaluating fish-habitat relations, fish distribution, and effects of dam removal in the Baraboo River, Wisconsin: University of Wisconsin-Stevens Point; 2002.

40. Catalano MJ, Bozek MA, Pellett TD. Effects of dam removal on fish assemblage structure and spatial distributions in the Baraboo River, Wisconsin. North American Journal of Fisheries Management. 2007;27(2):519-30.

41. Doyle MW, Stanley EH, Harbor JM. Channel adjustments following two dam removals in Wisconsin. Water Resources Research. 2003;39(1).

42. Riggsbee JA, Julian JP, Doyle MW, Wetzel RG. Suspended sediment, dissolved organic carbon, and dissolved nitrogen export during the dam removal process. Water Resources Research. 2007;43(9).

43. Sherman M. Potential impacts of small dam removal on fish and mussel communities in North Carolina: : Duke University; 2013.

44. Bushaw-Newton KL, Hart DD, Pizzuto JE, Thomson JR, Egan J, Ashley JT, et al. An integrative approach towards understanding ecological responses to dam removal: The Manatawny Creek Study. Journal of the American Water Resources Association. 2002;38(6):1581-99. doi: 10.1111/j.1752-1688.2002.tb04366.x.

45. Thomson JR, Hart DD, Charles DF, Nightengale TL, Winter DM. Effects of removal of a small dam on downstream macroinvertebrate and algal assemblages in a Pennsylvania stream. Journal of the North American Benthological Society. 2005;24(1):192-207.

46. Velinsky DJ, Bushaw-Newton KL, Kreeger DA, Johnson TE. Effects of small dam removal on stream chemistry in southeastern Pennsylvania. Journal of the North American Benthological Society. 2006;25(3):569-82.

47. Cui Y, Wooster JK, Braudrick CA, Orr BK. Lessons learned from sediment transport model predictions and long-term postremoval monitoring: Marmot Dam removal project on the Sandy River in Oregon. Journal of Hydraulic Engineering. 2014;140(9):04014044.

48. Keith MK. Reservoir evolution following the removal of Marmot Dam on the Sandy River, Oregon: Portland State University; 2012.

49. Podolak CJP. Channel bed response to an increased sediment supply: Johns Hopkins University; 2012.

50. Ferry M, Miller P. The removal of Saeltzer Dam on Clear Creek : an update. University of California, Berkeley: 2003.

51. Greenwald GM, Earley JT, Brown MR. Juvenile Salmonid Monitoring in Clear Creek, California, from July 2001 to July 2002. U.S. Fish and Wildlife Service, Red Bluff Fish and Wildlife Office, Red Bluff, California. : 2003.

52. Simons C, Walker K, Zimring M. Saeltzer Dam Removal on Clear Creek 11 years later: An assessment of upstream channel changes since the dam's removal. University of California: 2011.

53. Conlon M. A Hindcast Comparing the Response of the Souhegan River to Dam Removal with the Simulations of the Dam Removal Express Assessment Model-1: Boston College; 2013.

54. Pearson AJ, Snyder NP, Collins MJ. Rates and processes of channel response to dam removal with a sand‐filled impoundment. Water Resources Research. 2011;47(8).

55. Hogg RS, Coghlan Jr SM, Zydlewski J, Gardner C. Fish community response to a small-stream dam removal in a maine coastal river tributary. Transactions of the American Fisheries Society. 2015;144(3):467-79.

56. Lambing JH, Sando SK. Estimated loads of suspeneded sediment and selected trace elements transported through the Milltown Reservoir Project Area before and after the breaching of Milltown Dam in the Upper Clark Fork Basin, Montana, water year 2008. U.S. Geological Survey Scientific Investigations Report 2009-5095: 2009.

57. Sando SK, Lambing JH. Estimated loads of suspended sediment and selected trace elements transported through the Clark Fork basin, Montana, in selected periods before and after the breach of Milltown Dam (water years 1985-2009). U.S. Geological Survey Scientific Investigations Report 2011-5030: 2011.

58. Peck JA, Kasper NR. Multiyear assessment of the sedimentological impacts of the removal of the Munroe Falls Dam on the middle Cuyahoga River, Ohio. Reviews in Engineering Geology. 2013;21:81-92.

59. Rumschlag JH, Peck JA. Short-term sediment and morphologic response of the middle Cuyahoga River to the removal of the Munroe Falls Dam, Summit County, Ohio. Journal of Great Lakes Research. 2007;33:142-53.

60. Ahearn DS, Dahlgren RA. Sediment and nutrient dynamics following a low-head dam removal at Murphy Creek, California. Limnology and Oceanography. 2005;50(6):1752-62.

61. Schmitz D, Blank M, Ammondt S, Patten DT. Using historic aerial photography and paleohydrologic techniques to assess long-term ecological response to two Montana dam removals. Journal of Environmental Management. 2009;90:S237-S48.

62. Gibson AM, Villalobos LM, Snyder EB. Ecosystem responses to low-head dam removal: Assessment of physical habitat, water chemistry, and macroinvertebrates. Grand Valley State University: 2011.

63. Hirethota PS, Burzinski TE, Eggold BT. Changing habitat and biodiversity of the Lower Milwaukee River and Estuary. Wisconsin Department of Natural Resources: 2005.

64. Stanley EH, Doyle MW. A Geomorphic Perspective on Nutrient Retention Following Dam Removal. BioScience. 2002;52(8):693-701.

65. Conlon MD. Changes in water temperature following the removal of a low-head dam in Plymouth, Massachusetts: University of Massachusetts, Boston; 2015.

66. Cantwell MG, Perron MM, Sullivan JC, Katz DR, Burgess RM, King J. Assessing organic contaminant fluxes from contaminated sediments following dam removal in an urbanized river. Environmental Monitoring and Assessment. 2014;186(8):4841-55.

67. Magilligan F, Nislow K, Kynard B, Hackman A. Immediate changes in stream channel geomorphology, aquatic habitat, and fish assemblages following dam removal in a small upland catchment. Geomorphology. 2016;252:158-70.

68. Bowman SW. American Shad and Striped Bass spawning migration and habitat selection in the Neuse River, North Carolina: North Carolina State University; 2001.

69. Burdick SM, Hightower JE. Distribution of spawning activity by anadromous fishes in an Atlantic slope drainage after removal of a low-head dam. Transactions of the American Fisheries Society. 2006;135(5):1290-300.

70. Evans JE, Harris N, Webb L. The shortcomings of “passive” urban river restoration after low-head dam removal, Ottawa River (northwestern Ohio, USA): What the sedimentary record can teach us. Reviews in Engineering Geology. 2013;21:161-81.

71. Gottgens J. Impact of the Removal of the Secor Road Dam on the Fish Community Structure and Composition in the Ottawa River, Ohio. The University of Toledo, Toledo, OH: 2009.

72. Harris N. Sedimentological response of the 2007 removal of a low-head dam, Ottawa River, Toledo, Ohio: Bowling Green State University; 2008.

73. Tullos D, Cox M, Walter C. Sodom and Shearer Dam Removal Effectiveness Monitoring. Oregon Watershed Enhancement Board Grant #209-919-8740: 2013.

74. Pollard AI, Reed T. Benthic invertebrate assemblage change following dam removal in a Wisconsin stream. Hydrobiologia. 2004;513(1):51-8.

75. Harbold W, Stranko S, Kilian J, Ashton M, Graves P. Patapsco River Dam removal study: Assessing changes in American Eel distribution and aquatic communities. Maryland Department of Natural Resources: 2013.

76. Maloney KO, Dodd HR, Butler SE, Wahl DH. Changes in macroinvertebrate and fish assemblages in a medium‐sized river following a breach of a low‐head dam. Freshwater Biology. 2008;53(5):1055-68.

77. Cheng F, Granata T. Sediment transport and channel adjustments associated with dam removal: Field observations. Water Resources Research. 2007;43(3).

78. Granata T, Cheng F, Nechvatal M. Discharge and suspended sediment transport during deconstruction of a low-head dam. Journal of Hydraulic Engineering. 2008;134(5):652-7.

79. Krieger K, Stearns A. Response of the macroinvertebrate community of the Sandusky River in Seneca and Wyandot Counties, Ohio, to removal of the St. Johns Dam. National Center for Water Quality Research: 2007.

80. Nechvatal M. Effects of Dam Removal on Water Quality Variables: The Ohio State University; 2004.

81. Burroughs BA. Dam Removal Effects on Fluvial Geomorphology and Fish Populations, and Diet of Catostomids in the Pine River, Michigan: Michigan State University; 2003.

82. Burroughs BA. The rehabilitation potential of dam removal: temporal perspective from Michigan's past dam removals: Michigan State University; 2007.

83. Burroughs BA, Hayes DB, Klomp KD, Hansen JF, Mistak J. Effects of Stronach dam removal on fluvial geomorphology in the Pine River, Michigan, United States. Geomorphology. 2009;110(3):96-107.

84. Mistak J. Dam removal effects on fisheries resources, habitat, and summer diet of trout in the Pine River, Manistee County, Michigan: Michigan State University; 2001.

85. Stanley EH, Luebke MA, Doyle MW, Marshall DW. Short-term changes in channel form and macroinvertebrate communities following low-head dam removal. Journal of the North American Benthological Society. 2002;21(1):172-87.

86. Bulak J, Bettinger J, Kubach K, Leitner J, Marion C, Poly W, et al. Twelvemile Creek Dam removal monitoring: South Carolina. Department of Natural Resources: 2011.

87. Marion CA. South Atlantic stream fish assemblages: Multi-scale structuring factors, trait associations and channelization, and responses to dam removal: Clemson University; 2014.

88. Whitener PA. Twelve Mile Creek channel morphology, substrate composition, and the macroinvertebrate community changes resulting from multiple dam removals.: Clemson University; 2013.

89. Doucette TM. Local fish community structure before and after breaching of the Woolen Mills Dam, Rivanna River, Virginia: University of Virginia; 2009.

90. Kanehl PD, Lyons J, Nelson JE. Changes in the habitat and fish community of the Milwaukee River, Wisconsin, following removal of the Woolen Mills Dam. North American Journal of Fisheries Management. 1997;17(2):387-400.

91. Poulos HM, Miller KE, Kraczkowski ML, Welchel AW, Heineman R, Chernoff B. Fish assemblage response to a small dam removal in the Eightmile River system, Connecticut, USA. Environmental Management. 2014;54(5):1090-101.
