# Supplementary material for: Landscape context and the biophysical response of rivers to dam removal in the United States
Source: PLoS One. 2017 Jul 10;12(7):e0180107. doi: 10.1371/journal.pone.0180107 (PMC5503210; doi:10.1371/journal.pone.0180107)
Supplement: S2 Table — EPA Level I, II, and III Ecoregion classifications (https://www.epa.gov/eco-research/ecoregions-north-america) for dams listed in the National Anthropogenic Barrier Dataset (NABD), removed dams in the USGS Dam Removal Information Portal (DRIP), and removed dams with before- and after-removal studies (BAR). (DOCX) [file pone.0180107.s002.docx]

Supporting information Table 2. EPA Level I, II, and III Ecoregion classifications (https://www.epa.gov/eco-research/ecoregions-north-america) for dams listed in the National Anthropogenic Barrier Dataset (NABD), removed dams in the USGS Dam Removal Information Portal (DRIP), and removed dams with before- and after-removal studies (BAR).

| EPA Level I Ecoregion | EPA Level II Ecoregion | EPA Level III Ecoregion | # of existing dams in the NABD | # of removed dams in DRIP | # of BAR studies |
| --- | --- | --- | --- | --- | --- |
| Northern Forests | Atlantic Highlands | Northeastern Highlands | 1659 | 62 | 1 |
|  |  | North Central Appalachians | 287 | 23 | 0 |
|  | Mixed Wood Shield | Northern Minnesota Wetlands | 11 | 0 | 0 |
|  |  | Northern Lakes and Forests | 799 | 29 | 2 |
| Northwestern Forested Mountains | Western Cordillera | Cascades | 98 | 8 | 3 |
|  |  | Sierra Nevada | 216 | 2 | 0 |
|  |  | Eastern Cascades Slopes and Foothills | 144 | 4 | 2 |
|  |  | Columbia Plateau | 91 | 10 | 0 |
|  |  | Blue Mountains | 142 | 2 | 0 |
|  |  | Northern Rockies | 105 | 5 | 0 |
|  |  | Idaho Batholith | 82 | 4 | 0 |
|  |  | Middle Rockies | 315 | 9 | 2 |
|  |  | Wasatch and Uinta Mountains | 196 | 1 | 0 |
|  |  | Southern Rockies | 758 | 3 | 0 |
|  |  | Canadian Rockies | 15 | 2 | 0 |
|  |  | North Cascades | 34 | 3 | 0 |
|  |  | Klamath Mountains/CA High North Coast Range | 73 | 22 | 2 |
| Marine West Coast Forest | Marine West Coast Forest | Coast Range | 74 | 15 | 1 |
|  |  | Puget Lowland | 123 | 3 | 0 |
|  |  | Willamette Valley | 125 | 6 | 2 |
| Eastern Temperate Forests | Central USA Plains | Southeastern Wisconsin Till Plains | 219 | 36 | 3 |
|  |  | Central Corn Belt Plains | 207 | 23 | 2 |
|  |  | Eastern Corn Belt Plains | 437 | 15 | 4 |
|  |  | Huron/Erie Lake Plains | 52 | 9 | 0 |
|  | Mississippi Alluvial and Southeast USA Coastal Plains | Middle Atlantic Coastal Plain | 336 | 3 | 0 |
|  |  | Mississippi Alluvial Plain | 144 | 0 | 0 |
|  |  | Southern Coastal Plain | 301 | 2 | 1 |
|  |  | Atlantic Coastal Pine Barrens | 323 | 16 | 0 |
|  | Mixed Wood Plains | North Central Hardwod Forests | 503 | 26 | 2 |
|  |  | Driftless Area | 347 | 25 | 3 |
|  |  | Southern Michigan/Northern Indiana Drift Plains | 373 | 19 | 1 |
|  |  | Northeastern Coastal Zone | 1903 | 55 | 3 |
|  |  | Northern Allegheny Plateau | 585 | 6 |  |
|  |  | Erie Drift Plain | 355 | 21 | 1 |
|  |  | Acadian Plains and Hills | 316 | 24 | 2 |
|  |  | Eastern Great Lakes Lowlands | 325 | 7 | 1 |
|  | Ozark, Oachita-Appalachian Forests | Ouachita Mountains | 184 | 0 | 0 |
|  |  | Arkansas Valley | 388 | 0 | 0 |
|  |  | Boston Mountains | 47 | 0 | 0 |
|  |  | Ozark Highlands | 793 | 1 | 0 |
|  |  | Blue Ridge | 594 | 8 | 0 |
|  |  | Ridge and Valley | 995 | 110 | 1 |
|  |  | Southwestern Appalachians | 304 | 0 | 0 |
|  |  | Central Appalachians | 313 | 19 | 0 |
|  |  | Western Allegheny Plateau | 648 | 23 | 0 |
|  | Southeastern USA Plains | East Central Texas Plains | 488 | 0 | 0 |
|  |  | South Central Plains | 835 | 1 | 0 |
|  |  | Piedmont | 3929 | 23 | 3 |
|  |  | Northern Piedmont | 480 | 94 | 5 |
|  |  | Southeastern Plains | 5062 | 15 | 2 |
|  |  | Interior Plateau | 631 | 5 | 0 |
|  |  | Interior River Valleys and Hills | 940 | 1 | 0 |
|  |  | Mississippi Valley Loess Plains | 795 | 2 | 0 |
| Great Plains | South Central Semi-Arid Prairies | High Plains | 640 | 4 | 0 |
|  |  | Southern Tablelands | 665 | 1 | 0 |
|  |  | Central Great Plains | 3947 | 0 | 0 |
|  |  | Flint Hills | 850 | 1 | 0 |
|  |  | Cross Timbers | 1597 | 1 | 0 |
|  |  | Edwards Plateau | 267 | 0 | 0 |
|  |  | Texas Blackland Prairies | 594 | 2 | 0 |
|  | Tamaulipas-Texas Semiarid Plain | Southern Texas Plains | 416 | 0 | 0 |
|  | Temperate Prairies | Central Irregular Plains | 2019 | 0 | 0 |
|  |  | Northern Glaciated Plains | 391 | 1 | 1 |
|  |  | Western Corn Belt Plains | 2732 | 12 | 0 |
|  |  | Lake Agassiz Plain | 112 | 1 | 0 |
|  | West-Central Semi-Arid Prairies | Northwestern Glaciated Plains | 1012 | 2 | 0 |
|  |  | Northwestern Great Plains | 3664 | 8 | 0 |
|  |  | Nebraska Sand Hills | 47 | 0 | 0 |
|  | Western Gulf Coastal Plain | Western Gulf Plain | 123 | 0 | 0 |
| North American Deserts | Cold Deserts | Snake River Basin | 96 | 3 | 0 |
|  |  | Central Basin and Range | 289 | 0 | 0 |
|  |  | Wyoming Basin | 302 | 0 | 0 |
|  |  | Colorado Plateaus | 144 | 0 | 0 |
|  |  | Arizona/New Mexico Plateau | 137 | 1 | 0 |
|  |  | Northern Basin and Range | 206 | 2 | 0 |
|  | Warm Deserts | Mojave Basin & Range | 63 | 0 | 0 |
|  |  | Chihuahuan Deserts | 140 | 2 | 0 |
|  |  | Sonoran Basin and Range | 54 | 0 | 0 |
| Mediterranean California | Mediterranean California | Central California Foothills and Coastal Mountains | 295 | 14 | 2 |
|  |  | Central California Valley | 61 | 3 | 0 |
|  |  | Southern California Mountains | 43 | 2 | 0 |
|  |  | Southern California/Northern Baja Coast | 143 | 10 | 0 |
| Southern Semi-Arid Highlands | Western Sierra Madre Piedmont | Madrean Archipelago | 17 | 0 | 0 |
| Temperate Sierras | Upper Gila Mountains | Arizona/New Mexico Mountains | 139 | 2 | 1 |
| Tropical Wet Forests | Everglades | Southern Florida Coastal Plain | 68 | 0 | 0 |
